# Characterization and Control of the Microbial Community Affiliated with Copper or Aluminum Heat Exchangers of HVAC Systems

**DOI:** 10.1007/s00284-012-0137-0

**Published:** 2012-05-09

**Authors:** Michael G. Schmidt, Hubert H. Attaway, Silva Terzieva, Anna Marshall, Lisa L. Steed, Deborah Salzberg, Hameed A. Hamoodi, Jamil A. Khan, Charles E. Feigley, Harold. T. Michels

**Affiliations:** 1 Department of Microbiology and Immunology, Medical University of South Carolina, Charleston, SC USA; 2 Department of Pathology and Laboratory Medicine, Medical University of South Carolina, Charleston, SC USA; 3 Arnold School of Public Health, University of South Carolina, Columbia, SC USA; 4 Department of Mechanical Engineering, College of Engineering, University of South Carolina, Columbia, SC USA; 5 Copper Development Association, New York, NY USA

## Abstract

Microbial growth in heating ventilation and air-conditioning (HVAC) systems with the subsequent contamination of indoor air is of increasing concern. Microbes and the subsequent biofilms grow easily within heat exchangers. A comparative study where heat exchangers fabricated from antimicrobial copper were evaluated for their ability to limit microbial growth was conducted using a full-scale HVAC system under conditions of normal flow rates using single-pass outside air. Resident bacterial and fungal populations were quantitatively assessed by removing triplicate sets of coupons from each exchanger commencing the fourth week after their installation for the next 30 weeks. The intrinsic biofilm associated with each coupon was extracted and characterized using selective and differential media. The predominant organisms isolated from aluminum exchangers were species of *Methylobacterium* of which at least three colony morphologies and 11 distinct PFGE patterns we found; of the few bacteria isolated from the copper exchangers, the majority were species of *Bacillus.* The concentrations and type of bacteria recovered from the control, aluminum, exchangers were found to be dependent on the type of plating media used and were 11,411–47,257 CFU cm^−2^ per coupon surface. The concentration of fungi was found to average 378 CFU cm^−2^. Significantly lower concentrations of bacteria, 3 CFU cm^−2^, and fungi, 1 CFU cm^−2^, were recovered from copper exchangers regardless of the plating media used. Commonly used aluminum heat exchangers developed stable, mixed, bacterial/fungal biofilms in excess of 47,000 organisms per cm^2^ within 4 weeks of operation, whereas the antimicrobial properties of metallic copper were able to limit the microbial load affiliated with the copper heat exchangers to levels 99.97 % lower during the same time period.

## Introduction

In densely occupied buildings, airborne microbial contaminants can result in numerous adverse effects on human health and well-being, including inflammation and infections. Airborne bacteria and fungi have the potential to adversely impact human health by causing infections, allergic responses, or toxic effects. Thus microbial growth in heating ventilation and air-conditioning (HVAC) systems and subsequent contamination of the indoor air environment is of increasing concern. Pathogenic and toxin-producing fungi and bacteria thrive in dark, moist environments and the conditions in HVAC systems would appear to be ideal environments for the growth and propagation of microbes. Intrinsic microbial biofilms on air-handling exchanger coils are associated with lowered heat transfer efficiencies and increased corrosion [2], as well as potential odor issues [18, 19]. Thus, little or no growth of microorganisms on HVAC surfaces is optimally desired.

Microbial populations present in HVAC exchanger systems can be substantial. Researchers have reported bacterial concentrations up to 10^6^ CFU cm^−2^ on air-handling cooling coils [8]. Other researchers have shown that the automobile and household air conditioning units can discharge up to 2,500 CFU m^−3^ of bacteria and 1,000 CFU m^−3^ of fungi above ambient levels on initial startup [10]. The microbiological concentrations associated with the subsequent air stream 15–120 min after continuous use returned to background levels.

Trends in energy efficiency, having led to “tighter” buildings, have subsequently resulted in microbial contamination becoming a major source of reduced indoor air quality, sometimes referred to as “sick building syndrome”. According to the United States Environmental Protection Agency (EPA), contaminated HVAC systems can serve as a breeding ground for bacteria and fungi, and a substantial reservoir for viruses and fungal and bacterial spores. Numerous studies have documented microbial contamination of HVAC systems [20]. In many cases fungal, bacterial and other biological debris can serve as antigens resulting in the induction of allergic reactions and other immunological problems. Asthmatics in homes with air conditioning were found to have significantly higher rates of asthma symptoms than those in homes without air conditioning. While little research has been performed on the mechanisms associated with these adverse effects, it is clear that microorganisms are drawn into HVAC systems from indoor and outdoor air, and propagate on the surfaces within these systems. The growth of microbes in these environments requires the presence of nutrients, often resulting from the inherent dust collected by the system, and water. The most common source of liquid water in HVAC systems results from condensation of water vapor on coiling coils and fins during routine operation of the systems. The resulting condensate may be aerosolized from surfaces with subsequent deposition of the contaminants within in ducts or occupied indoor spaces.

Contaminants accumulate in HVAC systems on heat exchanger coils and fins, in condensate drain pans, on air filters, and in air ducts. Indoor surfaces and building occupants can then be exposed to bio-aerosols from these sites. Measurement of airborne fungi showed differences in concentrations and species diversity between adjacent regions of HVAC systems. Reduction of both total colony forming unit (CFU) counts and species diversity were greatest across the outdoor air intake. Further reduction was observed across the filters. Cooling coils also had substantial “filtration” effects. This demonstrates that significant fractions of viable airborne fungi are deposited within building HVAC systems [12].

A variety of commercial products on the market were designed to reduce biofilms and subsequent odors and health concerns associated with air conditioning systems. Some of these products have been tested under laboratory conditions and have demonstrated modest control of the growth of resident biofilms [3]. The inherent antimicrobial properties of copper and its alloys against both eukaryotic and prokaryotic organisms [4, 6, 16] offer an alternative approach to control the growth and distribution of pathogens and allergens through HVAC systems [22]. Uncoated copper surfaces are capable of killing bacteria, viruses, and fungi in very short periods of time. Pathogenic bacteria die within 90 min at room temperatures, and within a few hours as the temperature decreases [14, 15]. Similarly fungi and some viruses are killed within hours of being exposed to metallic copper surfaces [22]. Conversely, microorganisms have been found to survive for a month or more on surfaces made from stainless steel or aluminum.

The principal goal of this study was to evaluate whether or not the inherent antimicrobial properties of metallic copper, when substituted for aluminum found in heat exchangers, might limit the colonization and growth of microbes on HVAC systems and thereby improve indoor air quality.

## Methods

### System Parameters

**Fig. 1:**
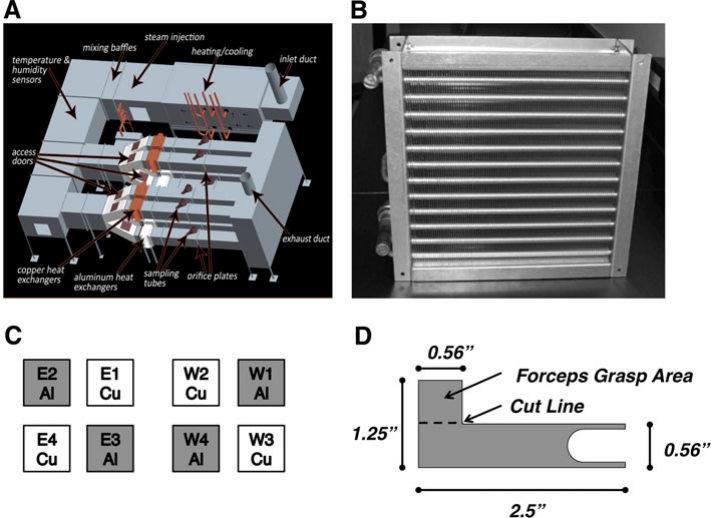
Schematic representation of full-scale HVAC test system used in this study. **a** General illustration of the test system; **b** Photographic representation of typical aluminum (control) heat exchanger (30.5 × 3.5 × 6.6 cm^3^ fin area dimensions) used in the full-scale HVAC test system; **c** orientation of copper and aluminum heat exchangers within the test system visualized from the perspective of the inflow side of the exchangers. *E* and *W* designations represent the east or west side of system; **d** schematic representation of the L-shaped coupons placed within each of the heat exchangers and retrieved weekly throughout the study. Coupons were manufactured from the same aluminum or copper alloy as the corresponding heat exchanger, were placed accordingly, and retrieved at selected time-points by grasping the top portion with sterile forceps. The coupons were deposited into sterile processing vials by cutting the upper L-portion of the coupon off with sterile scissors. The resident biofilm on each coupon was determined as described in the “Methods”

A full-scale test HVAC system, constructed at the University of South Carolina Public Health Research Center, was used to characterize the deposition and establishment of the microbial communities associated with copper and aluminum heat exchangers. The test system obtains its makeup air from the roof of the building, passes it through a primary heat exchanger where its temperature and humidity were adjusted to levels routinely encountered in air conditioning systems upstream of the heat exchangers. The delivery of this pre-conditioned makeup air was then sent to a parallel array of eight 1-ton capacity heat exchangers (Fig. 1a). Downstream of the heat exchangers, the air streams passed through Class II high efficiency, particulate, air (HEPA) filters (Flanders Pre-pleat 40, Washington, NC) and were subsequently discharged to the roof. The experimental heat exchangers, manufactured by Luvata Genada, LLC were made from either UNS A91100 aluminum or UNS C11000 copper and were 30.5 × 3.5 × 6.6 cm^3^ (12″ × 12″ × 2.6″ (finned area dimensions)) in a standard cross-flow, finned-tube configuration (Fig. 1b). Four copper and four aluminum exchangers were installed in the system in an alternating pattern (Fig. 1c). The system was operated, on average, at flow rates of 0.169–0.180 m^3^/s and incoming relative humidity and temperatures of 79 % and 27 °C, respectively. The exchangers were in “cooling mode” to mimic summer air-cooling conditions. Each exchanger was fed through its own ductwork containing similarly conditioned air.

### Biofilm Assessment

L-shaped coupons (Fig. 1d) were fabricated out of either aluminum or copper fin stock that was used to manufacture the corresponding heat exchangers. Ninety-nine sterile coupons were placed with the U-shaped portion of each coupon around the chilled-water tube. A plan for coupon placement and removal was developed to facilitate comparison of the microbial loads on the copper and aluminum coupons while balancing other variables which might exert some effect on loading. This removal plan was validated by numerical simulation assuming various differences between copper and aluminum coupon loadings. Aseptic removal of three coupons from each exchanger was performed on a weekly basis in order to assess the biofilm present. The L-shaped design of the coupon facilitated easy removal with sterile forceps and did not interfere with airflow or heat transfer. Upon removal each coupon was cut using sterile scissors in order to deliver the long untouched portion of the coupon into a sterile 50 ml conical tube. The resulting coupons were packaged and shipped overnight with refrigerant packaging to the Medical University of South Carolina, Charleston, SC for subsequent microbiological analysis.

### Microbiological Characterization

Coupon samples were aseptically bent inside the sample tube at a 45° angle in order to facilitate better mixing and extraction of the attached biofilm. Biofilm sampling was facilitated by adding ~20 sterile glass beads (2 mm dia.) to each tube containing a sample coupon along with 2 ml of phosphate buffered saline. The coupons were vortexed at high speed for 1 min. The resulting liquid was plated at various dilutions onto trypticase soy agar + cycloheximide (TSA + CE) (Hardy Diagnostics, Santa Maria, CA) for the assessment of viable bacteria as well as onto Sabouraud dextrose agar + chloramphenicol (SD + CL) (Remel, Lexana, KS) for the determination of viable fungi. Selected samples were also plated onto R2A agar (BD, Sparks, MD) + cycloheximide (50 mg/l) (R2A + CE) for comparison. Cycloheximide was incorporated into the bacterial plate media to inhibit fungal growth and conversely chloramphenicol was incorporated into the fungal medium to inhibit bacterial growth for purposes of better colony counting. The plates were incubated at 26 °C for 5–6 days and the colonies were enumerated. Data were reported as CFU cm^−2^ of coupon surface. Bacterial colonies were characterized using standard microbiological tests including gram stain, motility, growth on various selective growth substrates, as well as utilizing RapId NF Plus kit, an identification panel optimized for the characterization of oxidase-positive, gram-negative bacilli (Remel, Lexana, KS) and Biolog ID system (Biolog, Hayward, CA). Total fungi present on the test coupon surfaces were quantitated but not identified.

### Pulse-Field Gel Electrophoresis (PFGE)

Bacterial isolates isolated from the aluminum coupons characterized as *Methylobacterium* spp. were further analyzed using PFGE. The method of Gautom [5] for *Escherichia coli* was followed with the exception being modifications to the incubation times for; (1) cell lysis in the agar plugs, which was extended from 2 h at 55 °C to 18 h at 55 °C, and (2) restriction endonuclease digestion with *Xba*I. The time of restriction endonuclease digestion was extended from 2 h at 37 °C to 18 h at 37 °C.

## Results

**Fig. 2:**
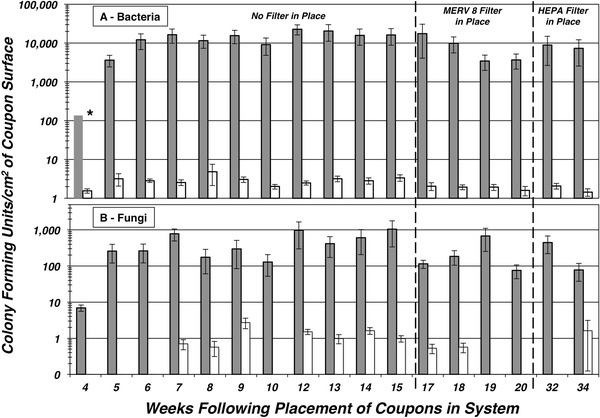
Intrinsic concentration of bacteria (**a**) or fungi (**b**) resident on copper (*white columns*) or aluminum coupons (*solid gray columns*) retrieved from their respective heat exchangers over a 30-week test period. Bacterial CFU cm^−2^ of coupon surface were determined from direct plate counts as assessed by growth on TSA + CE plates. Fungal CFU cm^−2^ of coupon surface were determined from direct plate counts as assessed by growth on SB + CL plates. The HVAC test system was initiated on week 1 and test coupons were inserted on week 3 with sampling beginning on week 4. The bacterial concentrations reported for week 4 exceeded levels that could reliably be determined using the dilutions used and were reported as >136 CFU cm^−2^

The HVAC test system was initiated with parameters associated with conditions used during the cooling season. Chilled water was circulated through the experimental heat exchangers. After 1 week of operation the system was temporarily stopped and 99 coupons of either copper or aluminum were installed into four equivalent copper and aluminum heat exchangers. Triplicate sets of coupons were removed from each exchanger commencing the fourth week after their installation. A robust biofilm was established on the aluminum heat exchanger within 4 weeks of system startup (Fig. 2). In contrast, the viable fungi and bacteria biofilms on the copper exchangers were at least one order of magnitude lower than the ones found on the aluminum exchangers. The average concentration of bacteria detected from the aluminum coupons was 11,411 CFU cm^−2^ and the values observed were consistently 3–4 orders of magnitude greater than the concentrations of bacteria recovered from the copper coupons, which averaged 3 CFU cm^−2^. The maximum and minimum concentration detected was 163,081 and 0 CFU cm^−2^, respectively. The range in the concentrations detected from copper coupons was significantly less with a maximum of 34 CFU cm^−2^ and a minimum of 0 CFU cm^−2^ being detected.

The concentration of viable fungi isolated from the aluminum coupons was lower than that of the bacteria recovered. The average coupon yielded 378 CFU cm^−2^ over the course of the 30-week study. Similar to the observations for bacteria, the fungal bioburden on the aluminum coupons was 2–3 orders of magnitude higher than that seen on copper coupons. The maximum fungal value recovered from aluminum coupons was 8,381 CFU cm^−2^ while the minimum was 0 CFU cm^−2^. The concentrations of fungi recovered from the copper coupons were on average <1 CFU cm^−2^ with maximum and minimums of 19 and 0 CFU cm^−2^, respectively.

The heat exchangers were initially supplied with unfiltered outside air that was pre-conditioned to a standard temperature and humidity level for the first 15 weeks subsequent to coupon placements in the system. In order to address the issue of whether or not the concentration of external microbes and particulates present in the unfiltered inlet air was serving as a source of nutrients or additional inocula for the established biofilm a minimum efficiency reporting value 8 (MERV 8) filter (Glassfloss Magna 1100 Series, Glasfloss, Dallas, TX) was installed, immediately upstream of the steam injection port (Fig. 1), on week 16. It was anticipated that the MERV 8 filter would capture particulate matter between 3 and 10 µm in size at efficiency of between 70 and 80 %. Placement of the filter should have served to reduce the particulate and fungal burden contacting the exchangers by ~70 % while allowing smaller air components such as free bacteria to pass through. While bacteria are generally smaller than 3 µm and fungi are predominantly larger, the majority of all airborne microbes are often associated with larger particles composed of plant matter, dust, and water. Thus, unless bacteria were present in the air stream as single cells, the MERV 8 filter should have also trapped a large fraction of the bacteria present in the air stream. The bacterial concentrations recovered from the aluminum coupons systematically dropped over the following 3 weeks and then stabilized (Fig. 2). This was presumably due to a reduction in the concentration of substrate previously supplied by the unfiltered makeup air. Similarly, a concomitant reduction in the number of fungi recovered from the aluminum coupons was seen within 1 week of the installation of the MERV 8 filter.

In a subsequent refinement, a HEPA filter was installed in place of the MERV 8 filter on week 24. The HEPA filter would remove particulates, bacteria, and fungi resident in the makeup air. The system was allowed to condition for 8 weeks at which times coupon extraction resumed. The bacteria and fungi recovered from samples taken on week 32 and 34 indicated that HEPA filtration had little to no effect on the established viable cells present within the biofilm resident on the coupons.

**Fig. 3:**
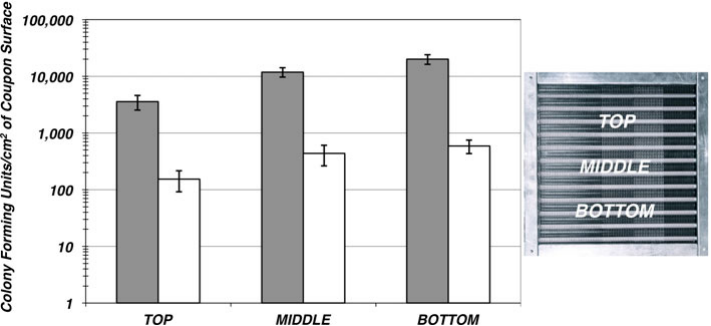
Comparison of the bacteria and fungi found on aluminum heat exchangers based on vertical location within the heat exchanger. Bacterial (CFU cm^−2^, *solid gray columns*) were determined from direct plate counts as assessed by growth on TSA + CE plates. Fungal (CFU cm^−2)^ were determined from direct plate counts as assessed by growth on SB + CL plates (*white columns*)

Quantitative analysis of the distribution of the viable bacteria and fungi within the heat exchangers revealed that the concentration of viable biomass was incrementally greater towards the bottom of the aluminum heat exchangers (Fig. 3). This was presumably due to the constant condensation formed on the fins washing the biofilm material downward through gravity. Similar analysis of the distribution of the viable bacteria and fungi within the copper exchanger revealed that the viable biomass was distributed uniformly through out the exchanger at concentrations <2 CFU cm^−2^.

**Fig. 4:**
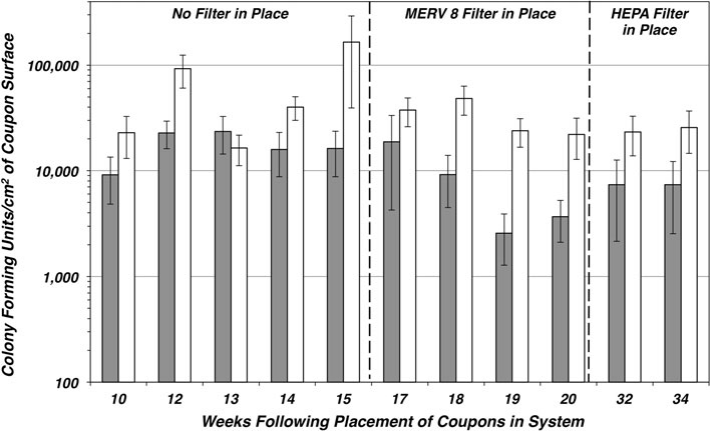
Recovery of viable bacteria from coupons is more efficient when using R2A + CE agar. The average concentration of bacteria (CFU cm^−2^) recovered from a subset of aluminum coupons was determined by plating each sample onto either TSA + CE agar (*solid gray columns*) or R2A + CE plates (*white columns*)

It was observed that the bacterial colonies routinely seen on the TSA + CE plates from the aluminum coupon samples were predominately small red colonies and to a lesser degree medium yellow colonies. The red colonies characteristically resembled members of the genus *Methylobacteria*. R2A medium, which was developed to study bacteria that are normally found to inhabit potable water and may be considered somewhat slow-growing or chemically stressed, is acknowledged as an appropriate substrate with which to isolate members of the genus *Methylobacteria* [17]. Ten weeks after sampling commenced, an additional sample from each coupon was plated onto R2A + CE plates. Supernatant from aluminum coupon samples plated onto this medium typically yielded red, pink, yellow, and to a lesser degree white bacterial colonies. Upon assessing the quantitative recovery of viable bacteria from the aluminum coupons it was recognized that plating samples onto R2A + CE yielded significantly higher concentrations of bacteria than when using the more nutritionally complex TSA + CE plates (Fig. 4). The average recovery from aluminum coupons increased using R2A + CE by 387 % during the evaluation period between weeks 10 and 34. The concentrations of viable bacteria associated with aluminum coupons from the 24 weeks of sampling using R2A + CE averaged 47,257 CFU cm^−2^ with a maximum concentration of 1,553,798 CFU cm^−2^ (data not shown). The viable bacteria recovered were consistently 4–5 orders of magnitude higher than the concentrations of bacteria recovered from the copper coupons, which averaged 3 CFU cm^−2^ (data not shown). Bacterial colony counts were very low on the copper coupon sampled and significant differences in recovery were not seen when using R2A + CE compared to TSA + CE. Of the few isolates recovered from copper coupons the majority were found to be aerobic, gram-positive, spore-forming rods and were classified as various members of the genus *Bacillus* sp.

The majority of the isolates recovered from the aluminum coupons plated on the R2A + CE medium appeared to belong to three distinct colony morphologies; (1) a dark red, small colony type, (2) a pink, small colony type, and (3) a pink, medium-sized colony type. Standard microbiological identification tests and visual observation including gram stain, morphology, biochemical analysis and growth on methanol indicated that each of the red and pink colonies tested were *Methylobacterium* spp. [7]. In addition to the red/pink *Methylobacterium* spp. colony types observed another colony morphology regularly isolated from the R2A + CE and TSA + CE plates was one that was medium sized and bright yellow. It was subsequently identified as *Sphingomonas* sp. The remaining colony types spanned a wide range and were white/tan in color and varied in both shape and size, tended to be gram-negative, and were not easily identified by the standard tests employed.

**Fig. 5:**
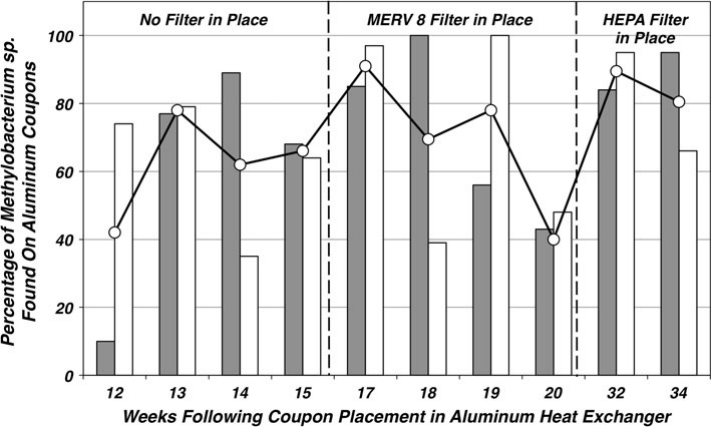
Representation of the population distribution of *Methylobacterium* spp. recovered from the selected aluminum coupons taken from the W1 and E3 heat exchangers during weeks 12–34. Bacterial CFU cm^−2^ of coupon surface were determined from direct plate counts as assessed by growth on R2A + CE plates. *Solid gray columns* represent the percentage of *Methylobacterium* spp. recovered from the total population of microbes isolated from the coupons recovered from E3 heat exchanger while the *white columns* represent the percentage of *Methylobacterium* spp. resident within the population of microbes isolated from the coupons recovered from the W1 heat exchanger. The average distribution is plotted as the *open circles*

A quantitative assessment of the number of methlyobacteria present in the biofilm associated with the aluminum heat exchangers was determined by counting the number of the total isolates recovered on the R2A + CE medium from coupons taken from the aluminum heat exchanger sites W1 and E3 between weeks 12 and 34 and then contrasting that number against the ratio of the number of red/pink colonies present within the sample. The average and individual percentage of *Methylobacterium* spp. represented in the population is described in Fig. 5. The fraction of *Methylobacterium* sp. resident in the biofilm population varied between the two samples with the variability being reflected in the average reported which was between 40 and 90 % of the recoverable population.

Analysis of thirty-four isolates from the three predominant methylobacteria colonies recovered from the R2A + CE medium was performed using PGFE. Evaluation of the electrophoretograms revealed that there were at least 11 distinct sub-strains present on the aluminum heat exchanger sites W1 and E3 (data not shown). This analysis, while not exhaustive, suggests that the methylobacteria resident within the established HVAC biofilms were diverse.

## Discussion

Significant quantitative and qualitative differences were observed between the microbes recovered from coupons taken from four copper and four aluminum heat exchangers in a test HVAC system over a 30-week course of sampling. Mixed biofilms, composed of bacteria and fungi, ranging in densities between 10^4^ and 10^6^ CFU cm^−2^ readily developed and were maintained on aluminum heat exchangers but were virtually non-existent on corresponding copper heat exchangers. The biofilms were most dense towards the base of the aluminum exchangers, presumably as a consequence of the gravitational distribution of condensate.

The type of medium used to measure the concentration of bacteria present on the coupons was found to be significant. A substantial increase in the number of viable bacteria recovered were observed when R2A + CE rather than TSA + CE was used as the primary plating medium for enumerating the viable bacteria present. Enhanced recovery using R2A + CE has been observed by others sampling liquid environments in which methylobacteria were isolated [1, 17, 21]. Enhanced recovery was not evident when plating samples obtained from the copper coupons.

The literature contains few descriptions of the microbiological makeup and concentrations of bacteria and fungi resident on the fins of heat exchangers. However, the data presented here are strikingly similar to the report of one group. Australian researchers reported bacterial concentrations of between 10^5^ and 10^6^ CFU cm^−2^ on air-handling cooling coils [8]. They derived their data using a swab/brush technique for the sampling of surfaces followed by enumeration on R2A plates. The data reported here from coupons recovered from aluminum exchangers were slightly lower but similar to what they observed. In addition, the predominant organism they isolated from the cooling coils was initially described as a *Blastobacter* sp. and later reclassified as a desiccation-resistant species of *Methylobacterium* [9] which was the same genus of the major type of bacteria recovered from the aluminum exchangers during the course of our study.

Others have similarly observed biofilms laden with methylobacteria from 10 of 11 test aluminum cooling coils used in automobile air conditioning systems obtained from three different countries [19]. In addition, a *Methylobacterium* spp./*Sphingomonas* sp. biofilm population was reported to be common on shower curtains [11]. A preliminary examination of a small number of isolates recovered by PFGE revealed that the population methylobacteria was diverse and was analogous to observations seen on shower curtains. The ecology of cyclical levels of high-moisture followed by extremes of desiccation on heat exchangers and shower curtains appears to be conducive to the establishment of biofilms with similar bacterial members.

The predominant bacteria isolated and identified from the aluminum systems were *Methylobacterium* spp. and *Sphingomonas* sp. Both sphingomonads, and to a lesser degree methylobacteria have been shown to cause infections, particular in individuals who are debilitated or immune-compromised [13]. Thus, the high concentrations of these organisms resident on the fins of the HVAC exchangers have the potential for discharge into the breathable air column air and likelihood of initiating health consequences.

The observation that of the small number of colonies recovered from the copper coupons were spore-forming bacilli leads one to question whether or not there were viable bacteria resident within the limited biofilm of the copper exchangers. These data may suggest that spores rather than vegetative cells were recovered from the copper coupons. Further work is required to substantiate this speculation.

The unfiltered air initially used as the makeup air for the non-sterile HVAC system likely served as the primary source of the inoculum that was responsible for the establishment of the respective biofilms resident on the four copper and four aluminum heat exchangers. The subsequent use of two filters, a MERV 8 and HEPA, within the system facilitated a limited reduction in concentration of viable bacteria recovered from the aluminum heat exchangers. However, the biofilms appeared quite resilient once formed and rebounded with continued operation of the system to concentrations seen prior to the installations of the filters. The source of nutrients available to the biofilm was not determined. It was possible that the expansive ductwork in the full-sized test system, collected dust and particulates over the course of operation and the turbulent flow of the air may have served to systematically dislodge sufficient organic and inorganic materials from which the biofilm resident on the moist surface of the heat exchanger could thrive. Conversely, the nutrients responsible for supporting the oligotrophic methylobacteria resident on the aluminum exchangers may have been gaseous and thus would easily pass through either of the filters employed.

It is acknowledged that while the complexities of the microbial communities resident on both types of heat exchangers warrant further study, the fundamental observation of this study was significant. Construction of heat exchangers from copper profoundly inhibited the concentration and diversity of microbes associated with the biofilms formed. The extent with which copper limited the growth of bacteria was 99.99 % and the limitation of fungal growth was found to be 99.74 % of that observed on the control, aluminum based heat exchangers. The data detailed here support the view of others [22] that, when copper is substituted for aluminum in the construction of the heat exchangers, a substantial and significant reduction in the biofilm associated with the heat transfer device found in residential and commercial HVAC systems can be achieved. Whether or not the subsequent reduction in burden will result in improvements to heat transfer and better indoor air quality is presently being addressed.
